# A study of the correlation between stroke and gut microbiota over the last 20years: a bibliometric analysis

**DOI:** 10.3389/fmicb.2023.1191758

**Published:** 2023-06-07

**Authors:** Shengnan Han, Longhui Cai, Peipei Chen, Weihong Kuang

**Affiliations:** ^1^Clinical Medical College of Acupuncture, Moxibustion and Rehabilitation, Guangzhou University of Chinese Medicine, Guangzhou, China; ^2^First School of Medicine, Guangzhou University of Chinese Medicine, Guangzhou, China; ^3^School of Medical Technology, Qiqihar Medical College, Qiqihar, Heilongjiang, China; ^4^Guangdong Key Laboratory for Research and Development of Natural Drugs, School of Pharmacy, Guangdong Medical University, Dongguan, China

**Keywords:** gut microbiota, stroke, bibliometric analysis, hotspots, research trends

## Abstract

**Purpose:**

This study intends to uncover a more thorough knowledge structure, research hotspots, and future trends in the field by presenting an overview of the relationship between stroke and gut microbiota in the past two decades.

**Method:**

Studies on stroke and gut microbiota correlations published between 1st January 2002 and 31st December 2021 were retrieved from the Web of Science Core Collection and then visualized and scientometrically analyzed using CiteSpace V.

**Results:**

A total of 660 papers were included in the study, among which the United States, the United Kingdom, and Germany were the leading research centers. Cleveland Clinic, Southern Medical University, and Chinese Academy of Science were the top three institutions. The NATURE was the most frequently co-cited journal. STANLEY L HAZEN was the most published author, and Tang WHW was the most cited one. The co-occurrence analysis revealed eight clusters (i.e., brain-gut microbiota axis, fecal microbiome transplantation, gut microbiota, hypertension, TMAO, ischemic stroke, neuroinflammation, atopobiosis). “gut microbiota,” “*Escherichia coli*,” “cardiovascular disease,” “risk,” “disease,” “ischemic stroke,” “stroke,” “metabolism,” “inflammation,” and “phosphatidylcholine” were the most recent keyword explosions.

**Conclusion:**

Findings suggest that in the next 10 years, the number of publications produced annually may increase significantly. Future research trends tend to concentrate on the mechanisms of stroke and gut microbiota, with the inflammation and immunological mechanisms, TMAO, and fecal transplantation as hotspots. And the relationship between these mechanisms and a particular cardiovascular illness may also be a future research trend.

## Introduction

Based on underlying neuropathology, strokes are classified as ischemic or hemorrhagic strokes (Parr et al., 2017). Ischemic strokes account for around 85% of all cases, whereas hemorrhagic strokes account for approximately 15%. Ischemic stroke is a form of brain injury caused by a brief or permanent blockage of blood flow to the brain, which can lead to severe neurological deficits, dementia, and even death (Hossmann, 2006). The World Health Organization declared in 2020 that stroke is a global health crisis and one of the most serious diseases of the twenty-first century. It is now the second leading cause of death in the world, following heart disease. Approximately 6 million people die annually because of stroke complications. Multi-system complications such as cardiac, respiratory, digestive, genitourinary, and thromboembolism are common, with up to 50% of patients experiencing gastrointestinal complications including constipation, dysphagia, gastrointestinal bleeding, and fecal incontinence (Schaller et al., 2006; Camara-Lemarroy et al., 2014; Ogata et al., 2014). The most prevalent causes of death following a stroke are lung and urinary tract infections, and the gut microbiota may be the source of systemic infection in stroke patients, particularly if the gut microbiota barrier is compromised and the immune system is suppressed after stroke (Chamorro et al., 2007). According to the published data, altered gut microbiota may be a risk factor for stroke and may influence stroke outcome (Murray and Lopez, 2013).

The human gut microbiota contains tens of trillions of bacteria, more than a thousand known types of bacteria, and around 3 million genes, which is 150 times larger than the human genome (Gill et al., 2006; Wekerle, 2017; Nam, 2019). The intestinal microbiome is controlled by only two phyla: Gram-Positive Microbes (e.g., Firmicutes) like Enterococcus and Lactobacillus, and Gram-Negative Bacilli (e.g., Bacteroides) (Eckburg et al., 2005; Mishra et al., 2016). Microorganisms and their metabolites in their normal physiological state provide key signals that help maintain human health. The gut bacteria influence the entire illness process, including the prognosis of stroke, through the synthesis of neurotransmitters (e.g., GABA), the generation of active metabolites (e.g., Short-chain fatty acids/SCFAs, TMAO), and the modulation of immune system activation (Sherwin et al., 2018). Stroke similarly causes gastrointestinal dysfunction, including reduced intestinal motility, increased permeability of the intestine, and dysbiosis of the gut microbiota (Braniste et al., 2014; Bermúdez-Humarán et al., 2019; Taché and Saavedra, 2022).

In contrast to other growing research disciplines, the link between gut microbiota and stroke is an emerging subject of the field, with the number of publications increasing from 11 in 2002 to 137 in 2021. Nevertheless, there is currently no study that objectively summarizes or analyzes the structure of knowledge and research trends in this significant topic. Bibliometrics is a crucial instrument for fully grasping the state of research in a particular scientific topic and employs a number of mathematical and statistical techniques to quantify data sets obtained from citation indexes (van Eck and Waltman, 2010; Chen et al., 2012). We will employ this strategy to attain the following goals: (1) utilize CiteSpace to measure the performance of research networks (e.g., countries, institutions, authors, journals, and disciplines, etc.) and analyze the achievements and gaps in research (Zhu S. et al., 2021). (2) Utilize CiteSpace to do clustering or developmental vector analysis of references and keywords, and to acquire the structure and believability of clusters, so as to assess the knowledge structure and evolution of research topics, as well as to predict trends in future research.

## Materials and methods

### Data collection

All data were extracted from the Web of Science (WOS) on 1st May 2022, including SCI-Expand, CCR-Expand, and IC.

### Search strategy

The data were collected using a combination of Medical Subject Headings (MeSH) and keyword searches. The search box was used to enter the subject terms “stroke” and “intestinal flora” as well as the corresponding entry terms. Further, we read the wider review literature to extract keywords, which was a supplement to the MeSH-based analysis. Following the aforementioned procedure, a first search of 748 original documents was carried out as shown in Table 1, then two researchers of this paper manually screened the remaining studies to remove any that were duplicated or irrelevant and kept the rest for further analysis. Controversial studies were handled by a third researcher. Six hundred and sixty records were exported in total.

**Table 1:** The topic search queries.

Set	Results	Search query
#5	748	#3 AND #4
#4	314983	TS = (Stroke OR Strokes OR Cerebrovascular Accident OR Cerebrovascular Accidents OR CVA (Cerebrovascular Accident) OR CVAs (Cerebrovascular Accident) OR Cerebrovascular Apoplexy OR Apoplexy, Cerebrovascular OR Vascular Accident, Brain OR Brain Vascular Accident OR Brain Vascular Accidents OR Vascular Accidents, Brain OR Cerebrovascular Stroke OR Cerebrovascular Strokes OR Stroke, Cerebrovascular OR Strokes, Cerebrovascular OR Apoplexy OR Cerebral Stroke OR Cerebral Strokes OR Stroke, Cerebral OR Strokes, Cerebral OR Stroke, Acute OR Acute Stroke OR Acute Strokes OR Strokes, Acute OR Cerebrovascular Accident, Acute OR Acute Cerebrovascular Accident OR Acute Cerebrovascular Accidents OR Cerebrovascular Accidents, Acute)
#3	587488	#1 OR #2
#2	584906	TS = (Microbiome OR Microflora OR Microbiota OR Flora OR Probiotic OR Saccharomyces OR Lactobacillus OR Bifidobacterium OR *Escherichia coli* OR dysbiosis)
#1	87905	TS = (Gastrointestinal Microbiome OR Gastrointestinal Microbiomes OR Microbiome, Gastrointestinal OR gut microbiota OR gut microbiotas OR Microbiome, Gut OR Gut Microflora OR Microflora, Gut OR Gut Microbiota OR Gut Microbiotas OR Microbiota, Gut OR Gastrogut microbiota OR Flora, Gastrointestinal OR gut microbiota OR Flora, Gut OR Gastrointestinal Microbiota OR Gastrointestinal Microbiotas OR Microbiota, Gastrointestinal OR Gastrointestinal Microbial Community OR Gastrointestinal Microbial Communities OR Microbial Community, Gastrointestinal OR Gastrointestinal Microflora OR Microflora, Gastrointestinal OR Gastric Microbiome OR Gastric Microbiomes OR Microbiome, Gastric OR Intestinal Microbiome OR Intestinal Microbiomes OR Microbiome, Intestinal OR Intestinal Microbiota OR Intestinal Microbiotas OR Microbiota, Intestinal OR Intestinal Microflora OR Microflora, Intestinal OR gut microbiota OR Flora, Intestinal OR Enteric Bacteria OR Bacteria, Enteric)

### Inclusion and exclusion criteria

Inclusion criteria: (1) published articles on stroke and gut microbiota, including original research articles, reviews, (2) publication year between 2002 and 2021, and (3) WOS as the citation database.

Exclusion criteria: (1) articles needing manual search or telephone access, papers not published in the public area, and multiple corrected papers were excluded, (2) articles unrelated to stroke and gut microbiota, (3) papers lacking a clear DOI, and (4) duplicate papers.

### Analysis tool

We use CiteSpace to analyze knowledge inflection points, research hotspots, evolutionary paths, knowledge structures, and emerging trends in knowledge domains, as well as to map the structure and dynamics of relationships between gut microbiota and stroke. In recent years, centrality analysis and highlighting functions have garnered considerable scholarly attention as essential indicators for identifying new trends. In addition, the identification of new trends based on CiteSpace’s calculated metrics does not necessitate the participation of subject-matter experts or prior familiarity with the issue.

**Figure 1:**
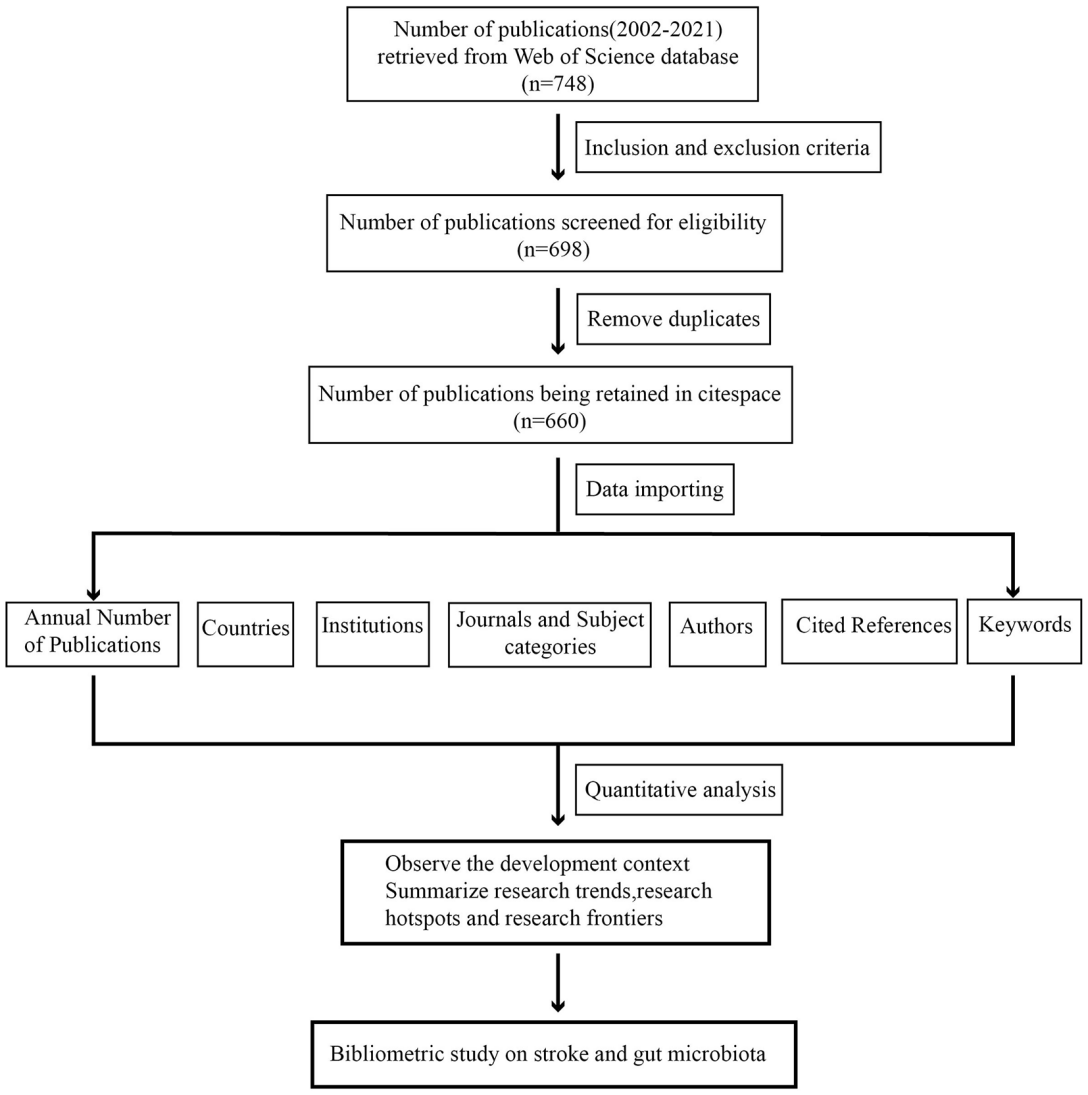
Flowchart of the inclusion of publications.

Our analysis’s procedures and components comprise the following three aspects: (1) descriptive statistical analysis: analysis of the annual publication, country, institution, journal, and author; (2) co-occurrence and co-citation analysis of the literature using the CiteSpace extension, as well as pulse analysis of research hotspots and dynamic evolution using references; and (3) keyword co-occurrence and emergent analysis. The detailed procedure is depicted in Figure 1.

Each node on a time slice is represented by a series of citation tree rings. The magnitude of the node shows how many citations obtained by the topic record. The hue of the nodes symbolizes the different time periods analyzed, with purple representing cited literature with high mediated centrality and red citation rings representing time slices with citation bursts or abrupt citation surges. Large clusters of citations with close relationships are generated by extracting the keywords and noun phrases from the titles of the citations in the cluster. The profile score of a cluster reflects its quality as an indicator of its homogeneity or consistency.

## Results and discussion

### Analysis of publications, nations, institutes, journals, and authors

**Figure 2:**
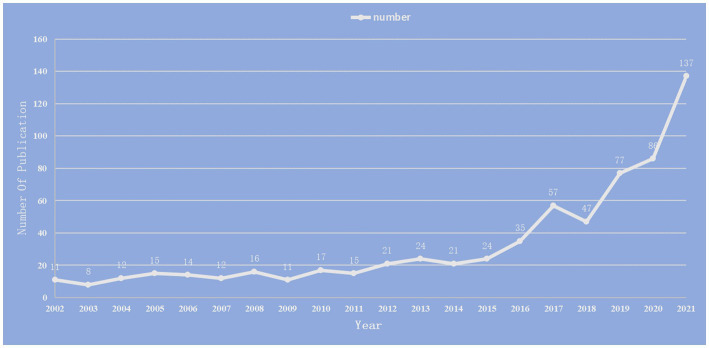
Annual number of publications on the study of the correlation between gut microbiota and stroke from 2002 to 2021.

We included 660 publications in total from January 1st, 2002, to December 31st, 2021. Research on the relationship between stroke and intestinal flora was analyzed in terms of annual publication, national distribution, institute distribution, journal distribution and author distribution. The total number of publications is on the rise, as indicated in Figure 2, with the biggest jump taking place between 2020 and 2021.

**Figure 3:**
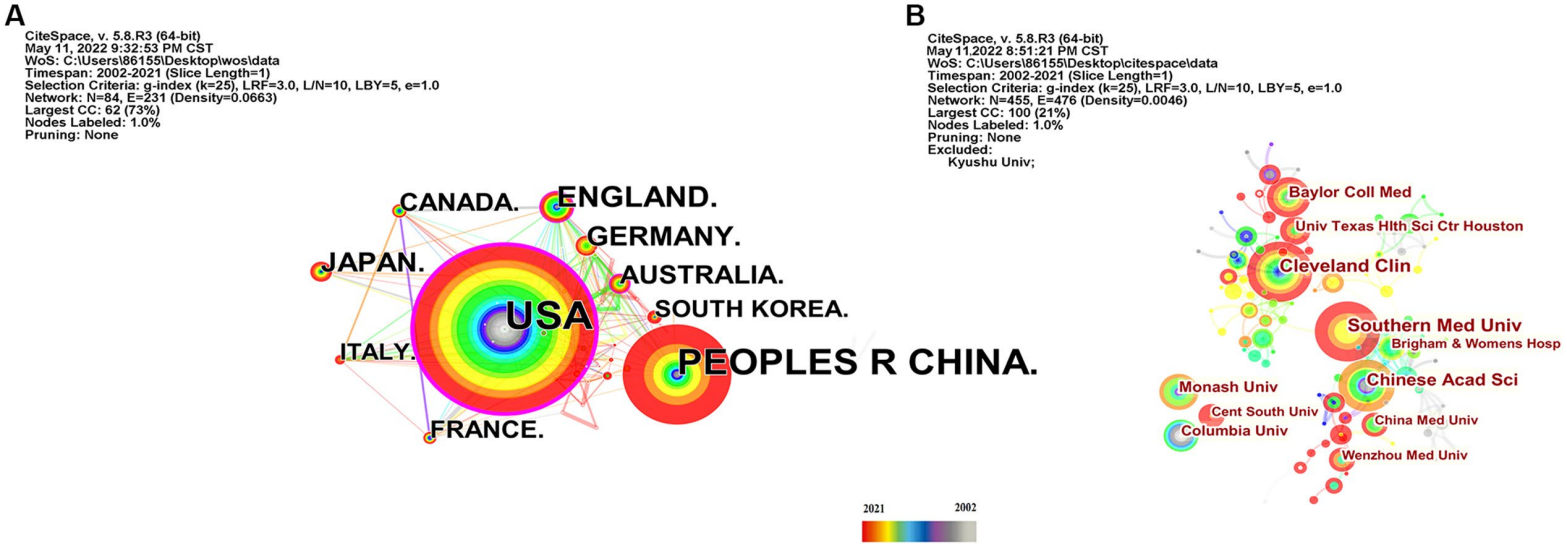
Country and institution map of the correlation between stroke and gut microbiota. **(A)** The network map of countries and regions for the correlation between stroke and gut microbiota. **(B)** Visualization of the correlation between stroke and gut microbiota by institutions.

As shown in Table 2, the USA topped the list in terms of total publications (220, 33.33%), single country publications (143, 29.92%), and intermediary centrality (0.53), followed by China and the UK. In addition, as depicted in Figure 3A, the outer circle in purple represents centrality, with larger circles signifying greater centrality. The United States (0.53) has the highest centrality, followed by the United Kingdom (0.2) and Australia (0.13).

**Table 2:** Top 10 countries/regions performed research on the study of the correlation between gut microbiota and stroke.

Countries	Rank	Total articles	Single-country articles	Rank and percentage (%)	internationally collaborative articles	Rank and percentage (%)
USA	1	220	143	1 (29.92)	83	1 (19.17)
China	2	134	122	2 (25.52)	37	3 (8.55)
England	3	46	10	10 (2.09)	39	2 (9.01)
Germany	4	35	18	4 (3.77)	24	5 (5.54)
Japan	5	35	33	3 (6.9)	13	8 (3)
Australia	6	29	14	7 (2.93)	26	4 (6)
Canada	7	26	16	6 (3.35)	15	7 (3.46)
France	8	24	12	8 (2.51)	16	6 (3.7)
South Korea	9	21	17	5 (3.56)	4	24 (0.92)
Italy	10	19	7	14 (1.46)	13	8 (3)

Scholars from 59 different countries contributed to this field of study. The top three institutions were Chinese Academy of Sciences (13, 1.74%), Southern Medical University (15, 2.01%), and Cleveland Clinic (15, 2.01%) (see Table 3). Inter-institutional contact can be made stronger in the future because the intermediate centrality is equal to 0. The Cleveland Clinic and Southern Medical University form two sub-networks with relatively low inter-institutional collaboration and relatively substantial intra-institutional collaboration, respectively, according to Figure 3B, which depicts the co-occurrence analysis of institutions. In 2013, The Cleveland Clinic published a study on trimethylamine-N-oxide (TMAO), an intestine microbial metabolite that contributes to atherosclerosis (Koeth et al., 2013). At Southern Medical University, more clinical studies have been carried out to offer stronger clinical proof of the association between dysbiosis of the gut flora and stroke (Yin et al., 2015; Zeng et al., 2019).

**Table 3:** Top 10 institutions performed research on the correlation between gut microbiota and stroke.

Rank	Freq	Centrality	Institution	Country
1	15	0.05	Cleveland Clinic	America
2	15	0.01	Southern Medical University	China
3	13	0.03	Chinese Academy of Science	China
4	10	0	Baylor College of Medicine	America
5	9	0	Monash University	Australia
6	8	0	Columbia University	America
7	7	0	University of Texas Health Science Center at Houston	America
8	6	0	Central South University	China
9	6	0	Wenzhou Medical University	China
10	6	0	China Medical University	China

**Figure 4:**
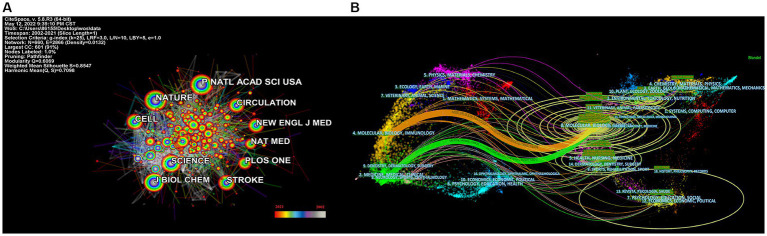
Categories and journal map of the correlation between stroke and gut microbiota. **(A)** Categories map of the correlation between stroke and gut microbiota from 2002 to 2021. **(B)** Co-citation journals map of the correlation between stroke and gut microbiota from 2002 to 2021.

The most often cited journal in a study of co-cited journals was NATURE (355), which was followed by NAS (312), PLOS ONE (273), STROKE (255), CELL (246) and SCIENCE (242), as is shown in Table 4 and Figure 4A. Additionally, we can see that the more specialized research subfields within the field are concentrated in the fields of cell biology, biochemistry, and molecular biology, indicating that the field is actually multidisciplinary, which is also consistent with the results of our double map of journals, Figure 4B.

**Table 4:** Top 10 relevant journals performed research on the correlation between gut microbiota and stroke.

Rank	Journal	Count	If (2021)	JCR category quartile
1	NATURE	355	49.962	MULTIDISCIPLINARY SCIENCES (Q1)
2	P NATL ACAD SCI USA	312	11.205	MULTIDISCIPLINARY SCIENCES (Q1)
3	PLOS ONE	273	3.24	MULTIDISCIPLINARY SCIENCES (Q2)
4	STROKE	255	7.914	PERIPHERAL VASCULAR DISEASE (Q1), CLINICAL NEUROLOGY (Q1)
5	CELL	246	41.582	CELL BIOLOGY (Q1), BIOCHEMISTRY AND MOLECULAR BIOLOGY (Q1)
6	SCIENCE	242	47.728	MULTIDISCIPLINARY SCIENCES (Q1)
7	NEW ENGL J MED	234	91.245	MEDICINE, GENERAL AND INTERNAL (Q1)
8	NAT MED	224	53.44	MEDICINE, RESEARCH AND EXPERIMENTAL (Q1), CELL BIOLOGY (Q1), BIOCHEMISTRY AND MOLECULAR BIOLOGY-(Q1)
9	J BIOL CHEM	216	5.157	BIOCHEMISTRY AND MOLECULAR BIOLOGY (Q2)
10	CIRCULATION	211	29.69	CARDIAC AND CARDIOVASCULAR SYSTEMS (Q1), PERIPHERAL VASCULAR DISEASE (Q1)

**Figure 5:**
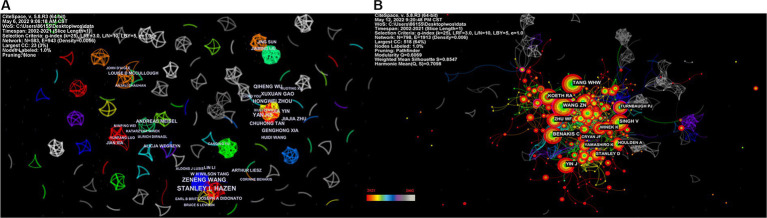
Network map of the correlation between stroke and gut microbiota by author and co-cited author. **(A)** Visualization map of main author cooperation network in the correlation between stroke and gut microbiota from 2002 to 2021. **(B)** Network visualization map of co-cited authors in the correlation between stroke and gut microbiota from 2002 to 2021.

Figure 5A depicts the authors’ collaboration network, which serves as a guide for locating research collaborators and discovering industry titans. According to Table 5, the top writers with the most publications were STANLEY L HAZEN (*n* = 12). A low mediated centrality of 0, indicating that these writers have had little impact on the topic and that additional research and collaboration are required. Finding research collaborators and industry leaders can be based on the author analysis and author co-citation analysis, which can help to suggest future research directions. As indicated in Figure 5B, Tang WHW (*n* = 108) is the most co-cited author, followed by Wang ZN (*n* = 102) with the greatest mediated centrality of 0.12. Despite receiving only 44 citations, Turnbaugh PJ’s publication has the highest mediated centrality of 0.12, indicating that it has provided a solid theoretical foundation for the subject.

**Table 5:** Top 5 authors and co-cited authors performed research on the correlation between gut microbiota and stroke.

Rank	Author	Freq	Co-cited author	Freq	Centrality
1	STANLEY L HAZEN	12	Tang WHW	108	0.02
2	ZENENG WANG	9	Wang ZN	102	0.12
3	YAN HE	7	Benakis C	99	0.03
4	J DAVID SPENCE	6	Singh V	89	0.01
5	QIHENG WU	6	Koeth RA	76	0.02

## Analysis of cited-references and keywords

**Figure 6:**
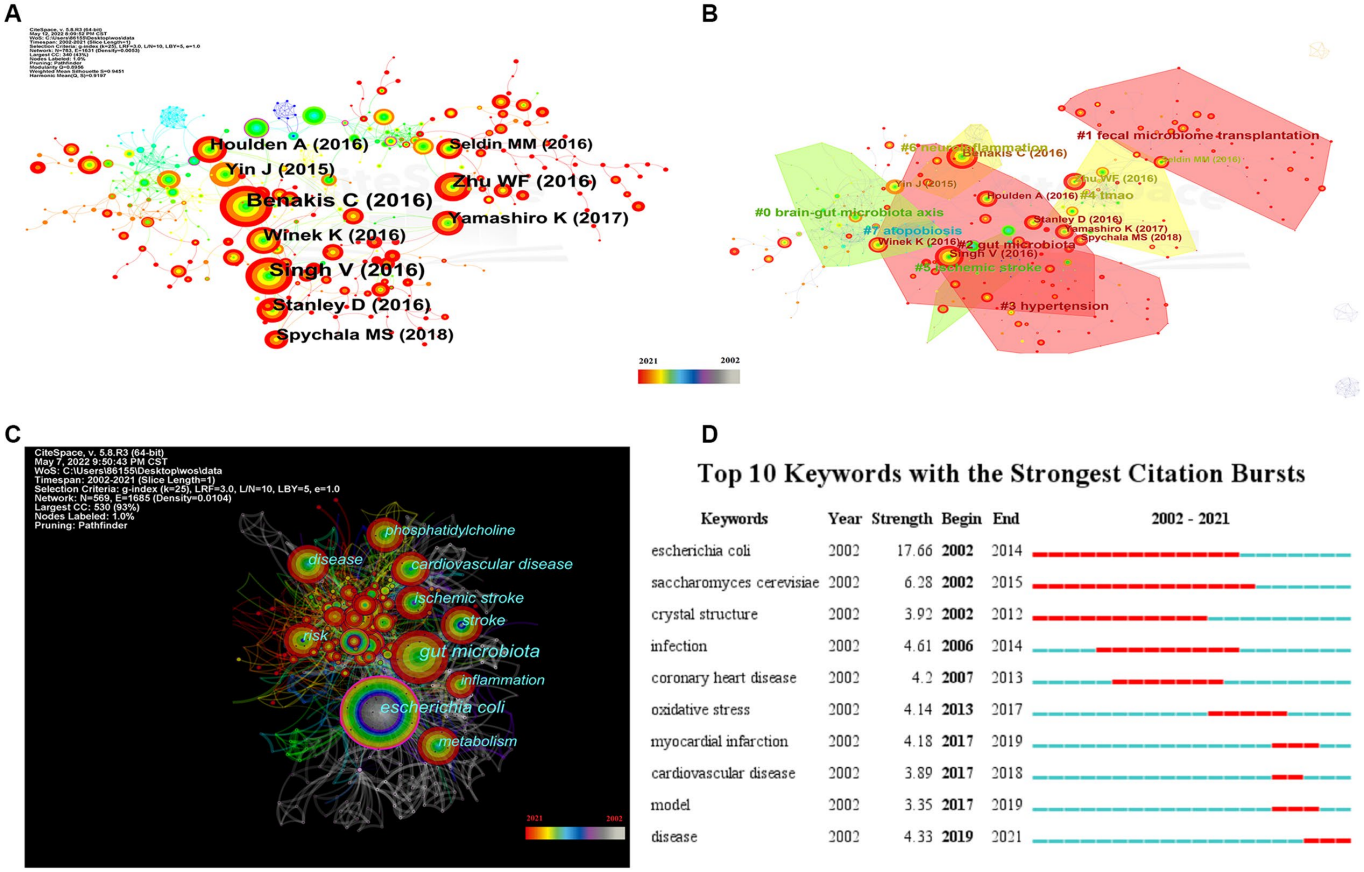
Visualization of keyword and reference for the correlation between stroke and gut microbiota. **(A)** The network map of cited reference related to the correlation between gut microbiota and stroke. **(B)** The co-occurrence network map of the cluster by reference for the correlation between gut microbiota and stroke. **(C)** Keyword co-occurrence network visualization of the research of the correlation between gut microbiota and stroke. **(D)** Top 10 keywords with the strongest citation bursts by CiteSpace.

**Figure 7:**
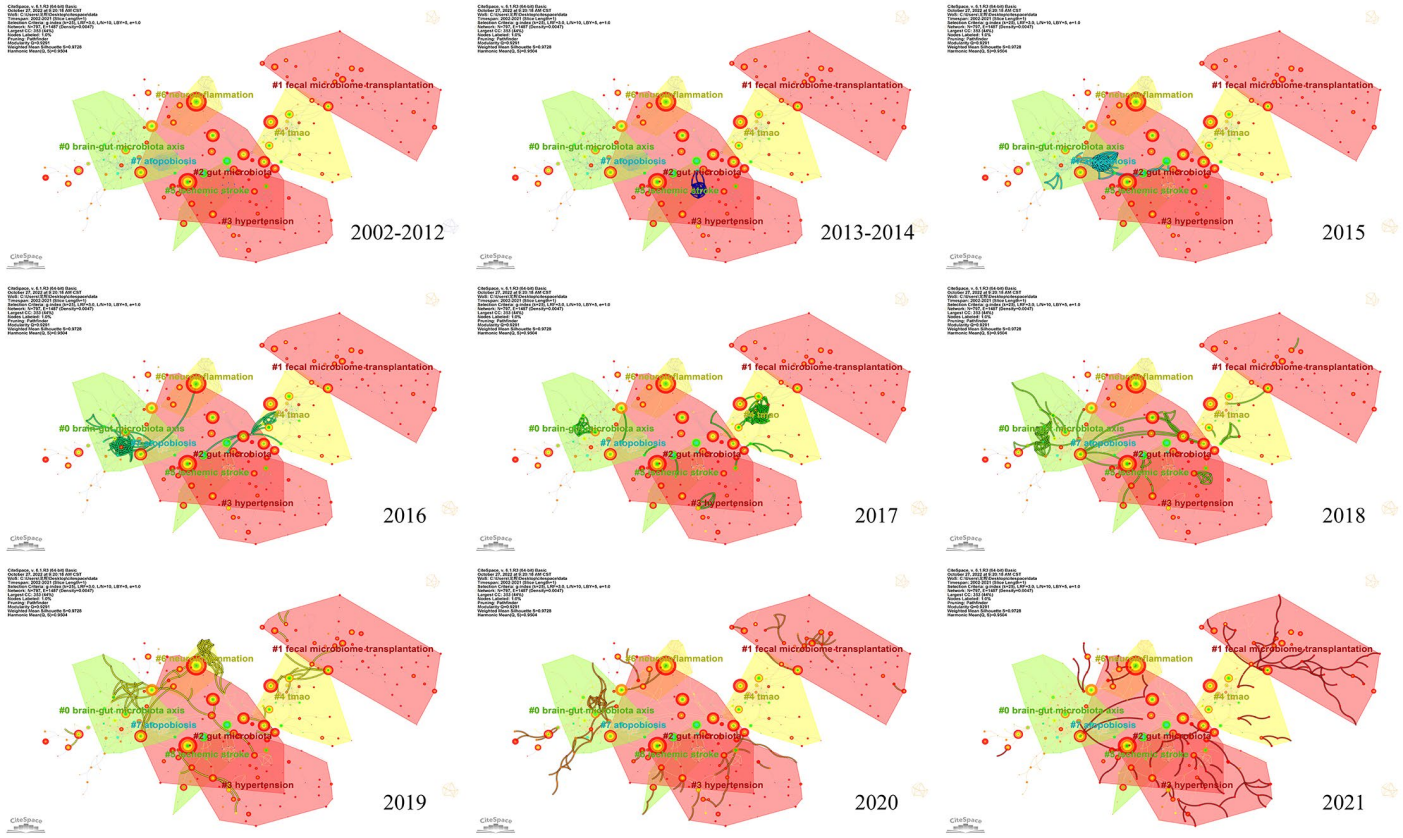
Research hotspots and dynamic evolution from 2002 to 2021.

To investigate the knowledge structure, research hotspots, and frontiers of the association between stroke and gut microbiota, we utilized CiteSpace to conduct co-occurrence and cluster analyses of references, as well as co-occurrence and emergent analyses of keywords for each publication in the database. Studies with a significant number of co-citations are sometimes regarded as the cornerstone of an area of study and can provide vital background information while representing the research goals of the community of basic scientists (Jones, 2016). Figure 6A displays a time slice of 1 for the period 2002 to 2021. The network diagram of the cited literature is comprised of 783 nodes and 1631 linkages, with the 50 most frequently mentioned or occurring records from each slice picked. As shown in Figure 6B, the members of the eight major clusters were as follows: #0 brain-gut microbiota axis, #1 fecal microbiome transplantation, #2 gut microbiota, #3 hypertension, #4 TMAO, #5 ischemic stroke, #6 neuroinflammation, and #7 atopobiosis. Cluster S ≥ 9.2 indicates high cluster homogeneity and consistency. Each cluster is denoted by a noun phrase derived from the keyword of the article cited in the cluster. Tang WHW (2013, 0.18), Karisson FH (2012, 0.17), Troseid M (2015, 0.15), and Wang ZN (2014, 0.11) possessed a high degree of intermediate centrality, who belonged to clusters #5 ischemic stroke, #0 brain-gut microbiota axis, #4 TMAO, and #4 TMAO, respectively. In addition, we analyzed the dynamics of the field (Figure 7), finding that there were no obvious hotspots of development in any particular subfield from 2002 to 2012, and hotspots of research in the areas of #2 gut microbiota, #3 hypertension, and #5 ischemic stroke from 2013 to 2014, with a shift in 2015 to the study of #5 ischemic stroke. In 2015, the focus shifted to the study of probiotics (#7 atopobiosis). In 2016, the focus shifted to the study of the brain-gut axis (#6 neuroinflammation). In 2017, a new subfield of research, TMAO (#4 TMAO), received a great deal of attention and research. From 2018 to 2021, the focus shifted back to the study of probiotics. During the period from 2018 to 2021, the research hotspots in this field were no longer confined to a single cluster, but had witnessed an extensive development of interconnections between clusters, most notably were the neuroinflammatory vein (#6 neuroinflammation) in 2019 and the fecal microbiome transplantation (#1 fecal microbiome transplantation) in 2021.

Figure 6C depicts the results of a co-occurrence analysis based on frequency for the top 50 keywords. Gut microbiota, *Escherichia coli*, cardiovascular disease, risk, disease, ischemic stroke, stroke metabolism, inflammation, and phosphatidylcholine are the 10 most prevalent phrases, as shown in Table 6. Burst words are key terms that have experienced a large increase in citations over a specific period and can be used to highlight research patterns over that time period. Execute CiteSpace with the “Burstterms” option to retrieve the burst graph keyword. As shown in Figure 6D, the words “*Escherichia coli*,” “*Saccharomyces cerevisiae*,” and “crystal structure” emerged between 2002 and 2014, demonstrating that research on gut microbiota has grown. Gut microbiota research was in its infancy, and scientists concentrated mostly on the species and structural composition of gut microbiota. “Infection,” “coronary heart disease,” “oxidative stress,” and “myocardial infarction” had been more common between 2006 and 2017. This indicates that scientists are beginning to investigate the processes underlying the association between gut microbiota and coronary heart disease or myocardial infarction. The terms “model” “cardiovascular disease” and “disease” appeared in the period 2017–2021, which, when combined with the hotspots and dynamics of the study in Figure 7, indicates that researchers have begun to focus on the links between specific disease mechanisms, such as hypertension, gut microbiota metabolites-TMAO, neuroinflammation, based on the progress made in the study of mechanisms related to gut microbiota and stroke.

**Table 6:** Top 10 co-occurrence keywords performed research on the correlation between gut microbiota and stroke.

Rank	Freq	Centrality	Keyword
1	123	0.04	Gut microbiota
2	92	0.54	*Escherichia coli*
3	58	0.08	Cardiovascular disease
4	56	0.05	Risk
5	56	0.05	Disease
6	53	0.08	Ischemic stroke
7	52	0.09	Stroke
8	48	0.02	Metabolism
9	40	0.04	Inflammation
10	39	0	Phosphatidylcholine

Based on the above bibliometric analysis of the references and keywords, we reviewed in detail the main areas of research on stroke and gut microbiota, including inflammation and immunity, TMAO, and fecal transplantation. It is worth noting that most of the examples used in this summary are derived from intermediate centrality or highly cited papers. Although the scope of our database is limited to the years 2011 to 2021, we have included instances from outside this period to illustrate the most notable improvements in each field, especially recently published works.

### Inflammation and immunity

About 70 percent of immune cells reside in the gastrointestinal (GI) tract, which maintains a balance between tolerability to the gut microbiota and immunoregulation (McDermott and Huffnagle, 2014). Inflammation and immunity are crucial components in the pathophysiology of stroke, involved in all phases of stroke, from pathogenetic mechanism of risk factors to neurotoxicity and then to tissue remodeling and repair (Iadecola and Anrather, 2011; Malone et al., 2019). Although the immunological response initiates in the ischemic brain parenchyma, the neuromediators produced *in situ* spread throughout the organism, resulting in a broad inflammatory reaction in the tissues (Anrather and Iadecola, 2016). In contrast, the gut microbiota is one of the targets of the systemic alterations generated by a stroke and has a significant impact on the result of stroke (Singh et al., 2016).

Intestinal microbiota dysbiosis is produced by an imbalance between commensal and pathogenic microbiota, with studies demonstrating an increase in proteobacteria and a decrease in Bacteroides in the feces of cerebral infarction patients (Wang et al., 2018). In contradiction to the above findings, the study found an increased abundance of Bacteroides 3 days after the mice had suffered an ischemic stroke and was considered to be characteristic of post-stroke ecological dysregulation (Singh et al., 2016). However, it is well-established that a large decrease in the species diversity of gut flora following a stroke is a major characteristic of post-stroke microbiome dysbiosis (Benakis et al., 2016; Yu et al., 2021).

The bidirectional MGBA balance is upset by post-stroke microbiota dysbiosis, which also exacerbates secondary brain injury and motor impairment. Translocation of the intestinal microbiota, dysbiosis of the intestinal flora (lower species diversity), and disruption of the tight junctions of the gastrointestinal tract in response to the stress of stroke. An unfavorable outcome, primarily in the form of an increase in the volume of cerebral infarction (Yuan et al., 2021).

#### Hyperactivation of the immune system

T cells play a decisive role in secondary neuroinflammation after cerebral ischemia and are key mediators of inflammatory collateral damage to the injured brain after stroke (Shichita et al., 2009; Neumann et al., 2015). T cell subsets include helper T cells (e.g., Th1, Th2, Th17) and regulatory T cells (e.g., Treg) (Zhen et al., 2016). Th1 excretes TNF-α, IFN-g, IL-12 and IL-2, and cellular factors IL-6, IL-17, IL-21 and IL-22 can be excreted from Th17, both of which activate the immune response (Halwani et al., 2017; Seyed and Rafati, 2021). Treg and Th2 relate to excreted cellular factors (e.g., IL-4, IL-10, IL-35, TGF-β) and have anti-inflammatory and brain-protective functions (Xu et al., 2020). The microbiota of the gut is a major modifier of T cell homeostasis (Ivanov et al., 2009). Dysbiosis of the gut microbiota produces pro-inflammatory Th17 and Th1 cell polarization (Singh et al., 2016).

**Figure 8:**
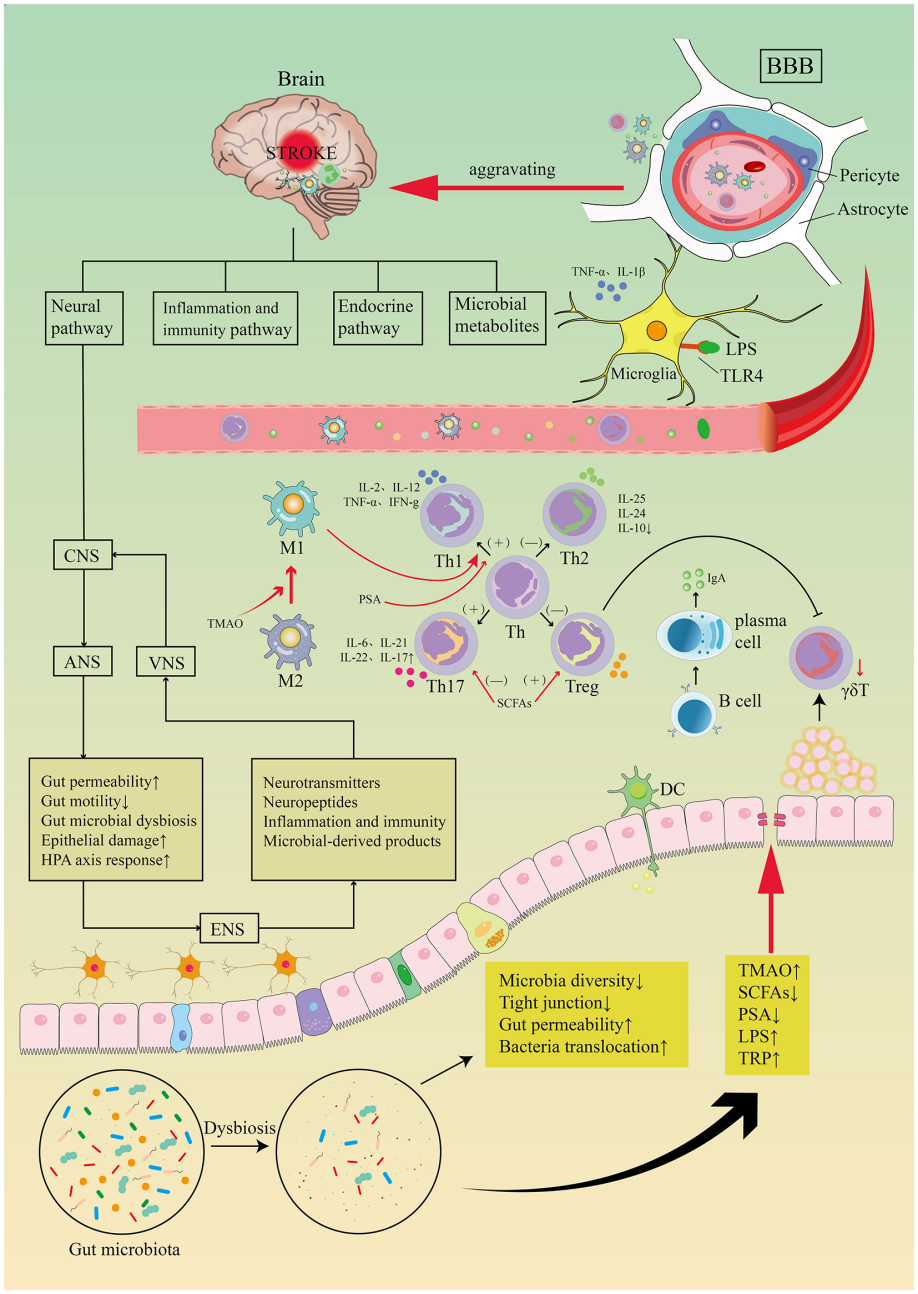
Image for potential association pathways between gut flora and stroke. The first is inflammatory and immune pathways. Stroke causes dysbiosis of the intestinal flora and decreased species diversity, impaired tight junctions and bacterial translocation, increased bacterial metabolites TMAO, LPS, TRP, decreased SCFAs, PSA. Intestinal flora regulates CD4+ T cell differentiation to CD8+ T cells via epithelial or DC cell-mediated signaling. T cell subsets can differentiate toward helper T cells (e.g., Th1, Th2, Th17) and regulatory T cells (e.g., Treg), the dysbiosis of the gut flora may trigger pro-inflammatory Th1 and Th17 Thelper cell polarization. Reduced manufacturing of γδT lymphocytes results in intestinal-to-membrane transfer, resulting to increased chemotactic factor production and brain infiltration of cytotoxic cells. Treg cells contribute in the post-stroke immunological cascade by inhibiting IL-17T cell proliferation via an intestinal route. SCFAs induce differentiation of naive T lymphocytes toward a functional Treg phenotype and suppress the pro-inflammatory Th17 phenotype, modulate microglia activation, and regulate synaptic plasticity after stroke. PSA also regulates differentiation of primitive CD4+ T cells toward Th1 cells, skewing the Th1/Th2 ratio in favor of Th1 cells. LPS stimulates Toll-like receptor 4 in brain microglia, astrocytes, and neurons, activating downstream immune responses and boosting the production of pro-inflammatory chemicals. TMAO enhances the polarization of M1 macrophages and the differentiation of Th cells into Th1 and Th17 subsets. Top-down neurotransmission mechanism: the sympathetic and parasympathetic nervous systems in the autonomic nervous system (ANS) integrate various messages from the brain, which are then transmitted to the enteric nervous system (ENS) or directly to the intestinal wall, resulting in necrosis and shedding of intestinal epithelial cells, increased intestinal permeability, impaired intestinal motility, and dysbiosis of the intestinal flora, HPA axis activation, and dysbiosis of the intestinal. Bottom-up neurotransmission mechanisms: a series of responses following intestinal flora disorders are perceived by the enteric nervous system (ENS) and reach the nucleus tractus solitarius (NTS) via sensory fiber inputs in the vagus nerve, which are then transmitted to extensive regions of the central nervous system for interpretation. Th, T helper; Treg, T regulatory; TMAO, trimethylamine-N-oxide; SCFAs, short-chain fatty acids; PSA, polysaccharide A; LPS, lipopolysaccharide; TRP, tryptophan.

An additional population of innate immune cells present in the gut epithelium is γδT cells (Prinz et al., 2013). Dysbiosis of the gut microbiota leads to a reduction in γδT lymphocyte production (Benakis et al., 2016). Pro-inflammatory IL-17 γδT cells recruited from intestinal tissue after stroke enhance the progression of cerebral ischemic injury (Gelderblom et al., 2012; Benakis et al., 2016). In animal models, activated T cells travel to the brain within 2 to 3 days following a stroke (Benakis et al., 2016). They are primarily found in the soft meninges and release IL-17 (IL-17 γδT cells), resulting in increased chemokine production and cytotoxic cell infiltration in the brain (e.g., neutrophils and monocytes). The anti-inflammatory cytokine IL-10 is excreted by regulatory T cells which decreases post-stroke inflammation and provide neuroprotection (Liesz et al., 2009). Increased amounts of Treg cells have been seen in the small intestine. But they do not enter the brain parenchyma. Post-stroke Treg cells appear to be engaged in the post-stroke immune cascade response by inhibiting IL-17 γδT cell proliferation via an intestinal route (Liesz et al., 2009). Benakis and colleagues (Benakis et al., 2016) found three putative methods of post-stroke microbial-brain communication: altered T-cell homeostasis, changed Treg/Th17 ratios, and intestinal lymphocytes migration to the cerebral ischemic. Utilizing a fluorescent labeling approach, Vikramjeet Singh and colleagues (Singh et al., 2016) were able to monitor locally labeled cells in the gastrointestinal tract. Fluorescently labeled T cells and monocytes were examined in the ischemic hemisphere after microinjection in the intestine 3 days after cMCAO, accounting for 25% of the overall T cell and Thelper cell subpopulation after cMCAO 3 days (see Figure 8). Together, these studies suggest a two-way relationship between the gut microbiota, the gut immune system, and the ischemic brain, where brain damage modifies the gut microbiota, which then affects stroke prognosis by regulating the post-ischemic inflammatory response.

#### Stimulation of bacterial metabolites

TMAO, SCFAs, PSA, LPS and tryptophan (TRP), among other microbial components and metabolites, are thought to cause neuroinflammation and modify the function of the central nervous system both immediately and by triggering the immigration of external immune cells to the cerebrum (Yuan et al., 2021). In addition to suppressing the pro-inflammatory Th17 phenotype and promoting the release of IL-10 by Th1 cells, SCFAs work in intestinal mucosal tissues to stimulate the development of naive T lymphocytes toward a functional Treg phenotype (Liesz et al., 2009; Sun et al., 2018; Sadler et al., 2020). SCFAs may also improve prognosis by enhancing tight junction proteins to protect intestinal epithelial cells from stroke-induced leaky gut. In laboratory stroke, a lower level of plasma SCFAs are associated with deteriorating outcome in mice with stroke, and replenishment of SCFAs recruits T lymphocytes and modulates microglia activation to improve post-stroke synaptic plasticity (Sadler et al., 2020). Toll-like receptor 2 (TLR2) on Treg cells and DCs is activated by PSA generated by *Bacteroides fragilis*, which causes the production of the anti-inflammatory cellular factor IL-10 (Zhang et al., 2017). Additionally, the development of early CD4^+^ T cells to Th1 cells can also be controlled by PSA, tilting the Th1/Th2 proportion in favor of Th1 cells (Krzyszczyk et al., 2020). By controlling T cells, dendritic cells, innate lymphoid cells, and macrophages, SCFAs will enhance anti-inflammatory cytokines (TGF-and IL-10) and decrease pro-inflammatory cytokines (TNF-α, IFN-γ, IL-12, IL-6, and IL-17A) (Arpaia et al., 2013; Honda and Littman, 2016; Thaiss et al., 2016).

The differentiation of helper T cell into Th1 and Th17 is mediated by polarized M1 macrophages that require NLRP3 inflammatory vesicles for activation. Studies have demonstrated that TMAO promotes the polarization of M1 macrophages (Wu K. et al., 2020). The macrophage phenotypes M1 and M2 are different from one another. The M1 macrophage subtype exacerbates areas of brain damage caused by IS and can potentially harm the central nervous system (Kwon et al., 2017), whereas the M2 macrophage facilitate blood supply reconstruction, wound recovery and organization construction (Barbay et al., 2015; Krzyszczyk et al., 2020). The scavenger receptor (SR), mannose receptor (MR), Arg-1, CD163, Fizz-1, and CD163 are surface membrane markers for the M2 macrophage subtype, and M2 can release the cytokines TGF-β and IL-10 (Kim et al., 2014; Yue et al., 2015). M1 macrophage subtypes may contribute to the generation of Th1. The pro-inflammatory M1 subtype of macrophages can replace the initial M2 subtype. M1 is pro-inflammatory, the major histocompatibility complex (MHC-II), CD16/32, CD40 and CD86 are located on the M1 isoform of the surface membrane of macrophages. These molecules cause the production of pro-inflammatory substances such TNF-α, IL-1b, IL-6, IL12, and IL-23 (Yang et al., 2022). The gut microbiome is essential for the activation and maturation of microglia, which can then be induced to become regulatory (M2) phenotype macrophages, reducing inflammation and promoting tissue repair (Ponomarev et al., 2013; Abdel-Haq et al., 2019).

Tryptophan is an amino acid that is metabolized by some microbiota and alters the function of immune cells in the gut. Increased tryptophan catabolism has been linked to worse stroke outcomes, according to previous studies (Jayaraj et al., 2019; Sadler et al., 2020).

#### Endotoxemia

Gram-negative bacteria’s outer membrane contains a significant amount of LPS, which readily crosses the compromised intestinal barrier into the bloodstream to cause metabolic endotoxemia (Cani et al., 2007). Through direct processes or by stimulating the immigration of external immune cells to the cerebrum, LPS can increase neuroinflammation and is a significant cardiovascular disease risk factor (Lukiw et al., 2018). Through the suppression of tight junction proteins caused by intestinal flora dysbiosis, intestinal permeability is increased, allowing lipopolysaccharide translocation into the bloodstream. LPS produced by intestinal dysregulation attach to Toll-like receptors (TLRs) and trigger a variety of immunological responses (Chacón et al., 2018). TLR 4 is the primary participant (Caso et al., 2007). Brain TLR4 receptors are stimulated by circulating LPS, and microglia, astrocytes, and neurons that express TLR4 are involved in stroke-induced brain injury (Gesuete et al., 2014). Lipopolysaccharides and TLR4 interact, activating the MYD88 and Nfκb pathways and increasing the production of pro-inflammatory cytokines (such as IL-6, IL-1, IL-27, and TNF-α). Atherosclerosis and cardiovascular disease (CVD) are both influenced by these inflammatory cytokines (Kwan and Hand, 2007; Vermeij et al., 2009). An intriguing study demonstrates that AIS in crab-eating monkeys causes long-term persistent elevation in plasma levels of LPS and pro-inflammatory cytokines that pass through the damaged intestinal barrier into the circulatory system, exacerbate neuroinflammation after cerebral ischemia, and contribute to the onset of type 2 diabetes by activating a pro-inflammatory cascade in adipose tissue (Cani et al., 2008; Chen Y. et al., 2019). According to a recent study, elevated plasma LPS levels were linked to worse stroke outcomes and had a positive correlation with the NIHSS and WBC counts in individuals who had ischemic strokes (Hakoupian et al., 2021). Additionally, patients with acute ischemic stroke have a worsened short-term prognosis when their plasma LPS levels are increased (Klimiec et al., 2016). Kurita et al. (2020) presented compelling evidence that metabolic endotoxemia is linked to poorer outcomes following stroke. After transient middle cerebral artery occlusion, type 2 diabetic (db/db) mice were raised with oral non-absorbable antibiotics; these mice had lower levels of inflammatory factors like TNF-α, IL-1β, and IL-6 and LPS than untreated db/db mice, which had a smaller cerebral infarct and increased neuroprotection. It is thought that chronic exposure to LPS may increase the neuroinflammatory response after stroke. Furthermore, it was discovered that (db/db) mice had an increased level of fecal Enterobacteriaceae, which is consistent with the findings of Geurts et al. (2011) and Yin et al. (2015). By way of the LPS-TLR4 pathway, Enterobacteriaceae overgrowth exacerbated cerebral infarction, according to research by Xu et al. (2021). Reducing nitrate production prevented Enterobacteriaceae overgrowth, decreased systemic inflammation, and further alleviated cerebral infarction. In the future, a novel potential treatment approach to enhance stroke outcomes may involve addressing metabolic endotoxemia.

#### Bacterial translocation and post-stroke infection

With rates ranging from 21 to 65%, infection and sepsis are significant consequences that affect stroke patients’ morbidity and mortality (Kwan and Hand, 2007; Vermeij et al., 2009). They have been shown to worsen stroke outcomes, extended inpatient treatment, and raise the risk of severe disability and even death (Hannawi et al., 2013; Li et al., 2014; Patel et al., 2020). According to several research, the severity of brain injury may be indicated by an infection (Vargas et al., 2006).

Most microorganisms found in patients with post-stroke infections were common commensal bacteria that normally inhabit the intestine. At present, many studies have shown that the causes of infection after stroke are intestinal barrier injury and BT (Tascilar et al., 2010). Using the middle cerebral artery occlusion (MCAO) mouse model, Tascilar et al. (2010) and colleagues performed the first animal study on bacterial translocation as a source of infection and analyzed the results of bacterial cultures from the lungs, spleen, liver, and jejunum of executed mice for the diagnosis of BT. Similar conclusions were drawn by Stanley et al. (2016) and Crapser et al. (2016). There must also be a function of the intestinal barrier, characterized by disruption of the tight junctions of the intestinal epithelium, for gut microbiota to disperse to the periphery (Hang et al., 2003; Doyle and Buckwalter, 2017). In addition to showing that increased intestinal barrier permeability makes it possible for gut microbiota to spread from the intestine to the outside, Shim and Wong (2018) also showed that following a stroke, the sympathetic nervous system (SNS) is responsible for producing increased intestinal permeability.

Bacterial translocation as a cause of systemic infection in stroke patients, particularly when host immunity and the immunological barrier are impacted by post-stroke immunosuppression (Latorre et al., 2015). Twelve hours after a stroke, the early immune system activation is replaced by a state of systemic immunosuppression, which lasts for many days. Reduced peripheral lymphocyte and T-cell reactivity, decreased monocyte activity, a rise in anti-inflammatory cytokines, lymphocyte death, splenic atrophy, and an expansion of regulatory T cells are all signs of immunosuppression (e.g., Treg) (Prass et al., 2003; Offner et al., 2006). Additionally, the SNS, an effector molecule that acts on adrenoceptors on immunocyte to promote immunosuppression, can be activated to cause post-stroke immunosuppression (Schulze et al., 2014). Studies have also been undertaken to investigate the possibility of adrenoceptor blockers to minimize infections associated with stroke have also been undertaken (Maier et al., 2015; Sykora et al., 2015). A retrospective study employing β-blockers to modify the SNS to preclude infection after apoplexy produced conflicting outcomes. However, to a lower degree than catecholamines, disruption of the HPA axis and consequent corticosteroid generation are also linked to apoplexy-derivative immune suppression (Prass et al., 2003; Mracsko et al., 2014). According to several research, immunosuppression brought on by a stroke was often severe by the fourth day and spontaneously transformed into a systemic bacterial infection during the first 3 days (Prass et al., 2003).

As a result of post-stroke infections, stroke patients are typically treated with antibiotics in the acute course of the disorder. Regarding the antibiotic treatment of post-stroke infections, there is considerable disagreement. Tang WHW and colleagues (Tang et al., 2013) suggest that the administration of antibiotics significantly suppresses fasting plasma TMAO levels, which rise again after antibiotic discontinuation. Christian Meisel (Winek et al., 2016) suggest that moxifloxacin prevents the development of infection and fever in stroke patients, significantly reduces mortality, and improves neurological prognosis. In contrast to this view, pretreatment with broad-spectrum antibiotics can lead to severe depletion of the microbiota, resulting in acute and severe colitis in experimental stroke. The PAT group selected fluoroquinolone and levofloxacin as the antibiotics of choice in a study of early systemic prophylaxis of post-stroke infections. When compared to the placebo group, levofloxacin prophylaxis not only failed to decrease the rate of infection during the 7 days after, but was also linked to worse stroke outcomes 90 days after the stroke (Chamorro et al., 2005). In comparison to untreated mice, *Streptococcus pneumoniae*-infected C57BL/6 mice showed greater mortality, more severe organ damage, increased pathogen dissemination, heightened inflammatory markers, and reduced alveolar macrophage function after antibiotic therapy, transferring fecal microbiota from animals not receiving treatment lowers lung bacterial populations and returns inflammatory markers to normal (Schuijt et al., 2016). Continuous antibiotic administration, according to Winek K, reduced the excessive mortality that occurred between days 5 and 7 following MCAO, shielded mice from colitis, increased survival, and improved cerebral ischemia outcomes while having no effect on infarct size at day 1 (Winek et al., 2016).

#### TMAO

Atherosclerosis is a chronic inflammatory condition marked by intimal thickening, proliferation of smooth muscle cells, migration and aggregation of monocytes and lymphocytes, platelet activity, and cholesterol accumulation (Gui et al., 2012; Drosos et al., 2015). It is the primary contributor to vascular disease globally, with ischemic stroke serving as its primary clinical manifestation (Herrington et al., 2016). TMAO, a phosphatidylcholine molecule generated by the liver enzymes and the gut microbiota, is thought to play a role in the etiology of numerous cardiovascular disorders (Wang et al., 2011; Haghikia et al., 2018). There is growing evidence that TMAO may enhance atherosclerosis (Wang et al., 2011; Stubbs et al., 2016). Possible mechanisms include enhanced vascular endothelial cell dysfunction and inflammatory injury, promotion of platelet activation and thrombosis, and inhibition of reverse cholesterol transport (Zhu et al., 2016).

#### TMAO’s mechanisms for promoting atherosclerosis

##### Vascular endothelial cell dysfunction and inflammatory injury

**Figure 9:**
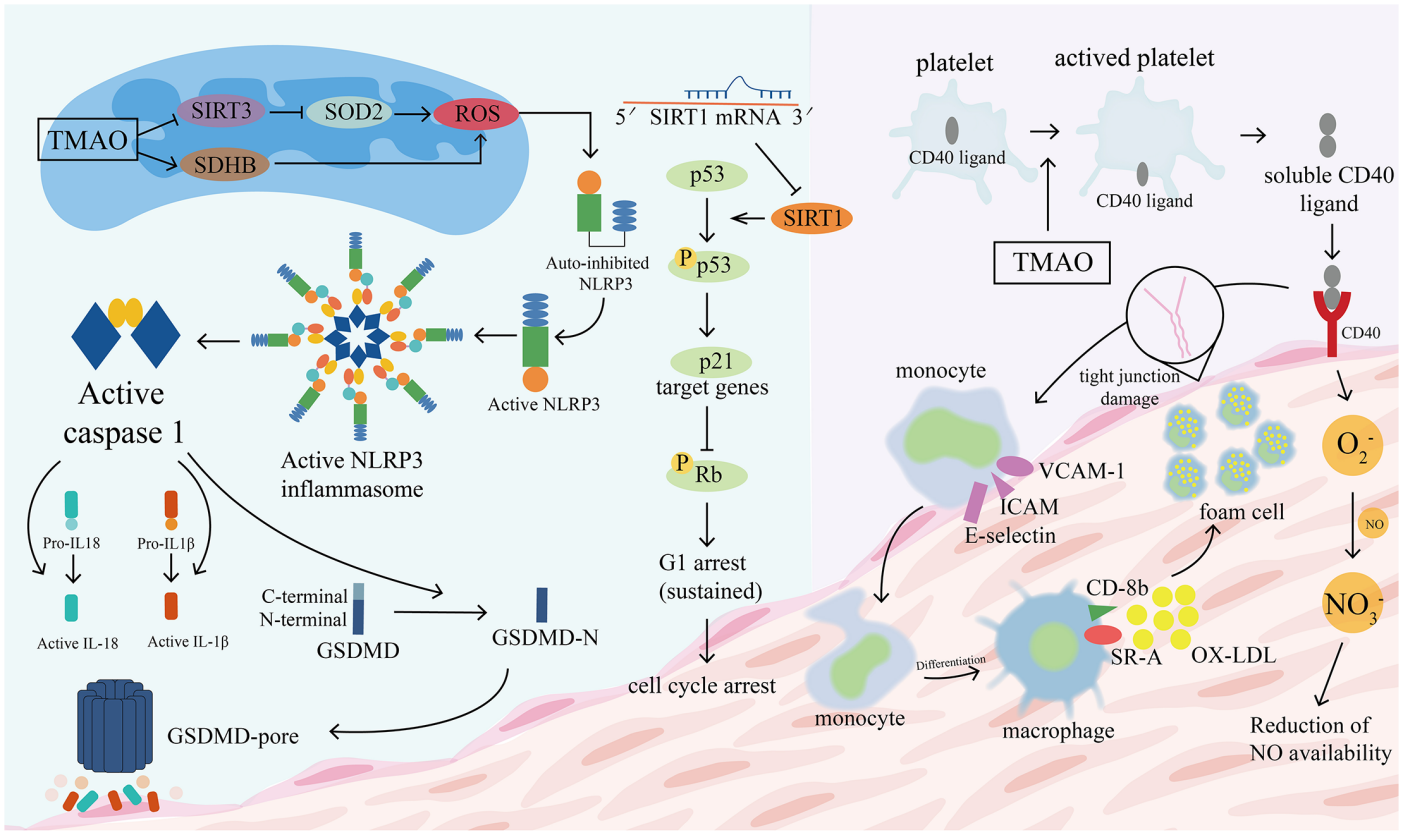
TMAO promotes the disruption of tight junction proteins in vascular epithelial cells. TMAO increases the expression of vascular cell adhesion molecule-1 (VCAM-1), promotes adhesion of monocytes and macrophages, and activates NLRP3 to stimulate the release of IL-18 and IL-1β. TMAO induces the activation of NLRP3, caspase-1, GSDMD-N and promotes GSDMD pore formation, leading to plasma membrane rupture, cell swelling and cytolysis. Two of the possible pathways that cause increased ROS are: the TMAO/SDHB/ROS pathway and the TMAO/SIRT3/SOD2/ROS pathway. TMAO inhibits Sirtuin 1 (SIRT1) activity and reduces cell growth by increasing the ratio of G0/G1 cell cycle through the p53/p21/Rb pathway. TMAO promotes platelet activation, with CD40 ligands located within platelets moving to the platelet surface to bind to CD40 located in the vascular epithelium, which triggers the following changes: (1) promotes the expression of inflammatory factors and adhesion molecules such as VCAM-1, ICAM, E-selectin, etc. (2) Promotes the conversion of monocytes to macrophages, which bind to ox-LDL to form foam cells. (3) Promotes the production of reactive oxygen species (O2-), which allows excessive NO consumption and impairs endothelial cell function. GSDMD, gasdermin D; VCAM-1, vascular cell adhesion molecule; ICAM, intercellular adhesion molecular; ROS, reactive oxygen species; SIRT3, deacetylase 3 (Sirtuin3); SOD2, superoxide dismutase 2.

Inflammatory damage to the vascular endothelium is usually considered to be the early stage of atherosclerosis and a key variable in its pathogenesis (Matsuzawa et al., 2015; Park and Park, 2015). The following processes are implicated (see Figure 9). (1) TMAO increases the expression of vascular cell adhesion molecule-1 (VCAM-1), which promotes the adhesion of monocytes and macrophages, and inhibits the ability of vascular endothelial cells to repair themselves, and decreases the expression of anti-inflammatory cytokines (which can make vascular endothelial cells more resistant to damage) (Ma et al., 2017). Multi-protein NLRP3 inflammatory vesicles stimulate the release of IL-18 and IL-1β and start an associated with inflammatory response (Martinon and Tschopp, 2007). Endothelial cell dysfunction is caused by TMAO because it causes the NLRP3 inflammasome to develop and become active. Researchers observed that TMAO markedly increased NLRP3 inflammatory vesicle activation, caspase-1 activity, release of the inflammatory cytokine IL-1β, and vascular endothelial cell permeability in carotid artery endothelial cells (CAECs) and partially ligated carotid arteries in wild-type mice (Boini et al., 2017). SIRT3-SOD2-mtROS signaling pathway activation may be one of the routes (Chen et al., 2017). The activity of protein kinase C (PKC) plays an important role in the regulation of endothelial dysfunction, including inflammation and adhesion (Durpès et al., 2015). In GuoHua Ma’s study (Ma et al., 2017), TMAO increased PKC activity in a dose-dependent manner compared to controls. In mice fed a choline-rich diet, high levels of TMAO in the study of Seldin et al. (2016) similarly increased inflammatory marker levels in aortic endothelial cells and smooth muscle cells and encouraged intravascular adhesion of leukocytes. They discovered that TMAO induced vascular inflammation by activating nuclear factor NF-κb and mitogen-activated protein kinase. The migration and multiplication of ECs may be essential for vascular self-repair (Xu, 2009; Pan et al., 2012). The current work demonstrates that TMAO decreases huvec proliferation and adherence to extracellular matrix (Ma et al., 2017). With its deacetylase activity, Sirtuin 1 (SIRT1) is an anti-aging molecule that targets numerous substrates (Kitada et al., 2016). The p53/p21/Rb pathway is activated by TMAO, which also reduces cell growth by increasing the ratio of the G0/G1 cell cycle and increasing oxidative stress (Ke et al., 2018). These findings suggest that high plasma TMAO limits vascular self-repair capabilities. (2) Encouraging the pyroptosis of vascular endothelial cells. Vascular endothelial cell pyroptosis is a pro-inflammatory form of programmed cell death. In contrast to apoptotic cells, the most important morphological changes in burned endothelial cells are loss of plasma membrane integrity and release of cell contents (Man et al., 2017). The formation of free GSDMD-N, which causes plasma membrane rupture, cell swelling, and cytolysis, is caused by the nucleotide-binding oligomeric structural domain-like receptor family member 3 (NLRP3)/gasdermin D (GSDMD) pathway (Lamkanfi and Dixit, 2012; Shi et al., 2015). TMAO dose-dependently induces NLRP3, caspase-1, GSDMD-N, and IL-18 protein expression, which significantly reduces ALDH2 activity and promotes a senescent (Nannelli et al., 2018; Li et al., 2022). Another mechanism is the SDHB/ROS pathway, which is activated when TMAO damages mitochondrial structure and function by upregulating SDHB expression and increases ROS production, promoting endothelial cell pyrolysis and subsequent release of pro-inflammatory cytokines (Wu P. et al., 2020). (3) Vascular endothelial cells become more permeable as the tight junctional integrity of the endothelium is compromised. The tight junction protein ZO-1 is expressed less frequently in EC monolayers after being stimulated with TMAO, which also changes the ECs’ permeability (Boini et al., 2017). TLR-4 is present on the surface of a variety of inflammatory cells, including vascular endothelial cells, and its binding to HMGB1 stimulates the body’s inflammatory response. The upregulation of HMGB1 by TMAO can exacerbate vascular inflammation (Singh et al., 2019).

By deactivating MAPK signaling, naringin restores the endothelium’s structural and functional integrity (Zhao and Zhao, 2022). Leukocyte adhesion and the inflammatory response were decreased as a result of apigenin’s ability to counteract TMAO’s effects on the adhesion molecule ICAM-1 and the inflammasome protein NLRP3 (Yamagata et al., 2019). The proliferation of endothelial cells was enhanced by asparagus extract (AE) (Wu et al., 2019). A TMA synthesis inhibitor called 3,3-dimethyl-1-butanol (DMB) decreased levels of circulating TMAO and improved age-related declines in EDD and eNOS expression, potentially offering new therapeutic approaches for the prevention and treatment of vascular senescence and CVD (Li T. et al., 2017; Ke et al., 2018).

##### Platelet activation and thrombosis

We analyzed the highly relevant citations based on four co-cited references with high mediated centrality. The most influential one of these was ZHU W’s article (2016) in Cell titled “The gut microbiota metabolite TMAO increases platelet hyperresponsiveness and thrombotic risk,” which illustrated a mechanistic connection with both TMAO and stroke that was previously unknown. Thrombosis is facilitated and platelet hyperresponsiveness is modulated by TMAO. He also pinpoints particular microbial taxa connected to TMAO and thrombosis, which was innovative and difficult (Zhu et al., 2016).

There is scant and circumstantial evidence supporting the platelets’ significance in human atherosclerosis. Platelet activation and aggregation are crucial processes in atherosclerotic thrombosis and have a major impact on the evolution of this step, according to existing research (Tomlinson and Wheeler, 2017; Roberts et al., 2018; Skye et al., 2018). ADP, prothrombin, epinephrine, and thromboxane a2 in the blood maintain the initial platelet response. When inflammation stimulates the local vascular endothelium, it increases the secretion of the platelet glycoprotein Ib ligand Von Willebrand, which encourages strong platelet adherence to the inflammatory site and aids in the growth of persistent atherosclerotic plaques. These lesions can burst and cause sudden attacks of arterial thrombosis (Davì and Patrono, 2007). TMAO can produce IP3 by decomposing platelet membrane phospholipids, which triggers the release of intracellular Ca^2+^ from internal storage, and leads to platelet activation (Zhu et al., 2016). When platelets are activated, CD40 ligands, which were previously present inside the cells when the platelets were at rest, emerge on the platelet surface and are broken down into soluble CD40 fragments. These fragments then bind to CD40 in vascular epithelial cells and encourage the expression of tissue factors and leukocyte adhesion factors (Denis et al., 2005). Examples include platelet-derived growth factor (PDGF), vascular endothelial growth factor (VEGF), and P-selectin (CD62P), which stimulate the release of chemokines from monocytes and macrophages and promote the differentiation of monocytes toward macrophages (Zhu et al., 2018). Substantial local leukocyte recruitment, modification of endothelial cell chemotactic and sticky characteristics, and worsening of atherosclerosis in apoe−/− animals (Tang et al., 2015). Large granules released by platelet membranes contribute to thrombosis by obstructing blood flow and creating a pro-thrombotic condition (Duhamel et al., 2007). Reactive oxygen species, when bound to ON, hinder the late disintegration of the generated thrombus (Williams et al., 2005). Reactive oxygen species are produced and released by vascular epithelial cells as a result of activated platelets. On the surface of macrophages, sR-A and cluster of differentiation 36 (CD36), a series of pattern recognition receptors (PRRs), recognize and phagocytose oxidized low density lipoproteins (ox-LDL) (Yang et al., 2019). Moreover, TMAO activates them, which encourages the production of foam cells and lipid buildup (Wang et al., 2011; Marcovecchio et al., 2017). Moreover, TMAO encourages the growth of vascular smooth muscle cells, which contributes to atherosclerosis formation (Duhamel et al., 2007).

Additionally, TMAO greatly increased the formation of reactive oxygen species (ROS), suppressed eNOS mRNA and protein expression, and promoted the scavenging of NO. Increased lipid peroxidation of circulating LDL cholesterol or cell membrane phospholipids can be caused by an increase in reactive oxygen species, activating processes that regulate platelet adhesion (Morrow et al., 1990; Praticò et al., 1996). In summary, increased TMAO levels in the blood are linked to lower eNOS-derived NO generation, oxidative stress, and an active inflammatory response, which in turn contribute directly or briefly to vascular endothelial cell failure (Li et al., 2018). Research on TMAO can help us better understand the processes that connect inflammatory mediators to platelet activation, which will lower the prevalence of CVD.

##### Inhibition of reverse cholesterol transport

High blood cholesterol levels, also known as hypercholesterolemia, have a pathogenic role in the progression of atherosclerosis and are one of the most significant dangerous factors for the cardiovascular disease, causing myocardial infarction and stroke (Mortensen and Nordestgaard, 2020). Following endothelial damage, Ross and Glomset (1973) originally hypothesized that atherosclerotic lesions develop when there was an unbalance between the deposition and clearance of arterial cholesterol (Sun X. et al., 2016). This suggested there was a link between Reverse cholesterol transport (RCT) and AS and reverse cholesterol transport was hindered by TMAO (Sun X. et al., 2016). RCT involves moving extra cholesterol to the liver and small intestine to prevent the build-up of extra cholesterol in peripheral tissues (Chistiakov et al., 2015), the key mechanism being macrophage efflux for liver excretion. Revolving LDL granules bind immediately to endothelial scavenger receptor type b1 (SR-B1), increasing the development of foam cells by mediating its transcellular transit and distribution to the subendothelial extracellular matrix for oxidation (as macrophages internalized oxidized LDL particles, causing them to become foam cells). TMAO upregulates the expression of cluster of differentiation 36 (CD36) and scavenger receptors located on macrophages, leading to the accumulation of immune cells and cholesterol and promoting atherosclerosis (Geng et al., 2018). ABCG1 mediates cholesterol efflux from macrophages, preventing foam cell formation (Hardy et al., 2017). ABCA1 mediates the first step in reverse transport, leading to a large accumulation of cholesterol in macrophages, and TMAO downregulates ABCG1 expression in macrophages (Koeth et al., 2013; Collins et al., 2016; Zhao et al., 2019). Additionally, the main pathway for cholesterol elimination is the metabolic synthesis of bile acids in the liver (Zárate et al., 2016). Studies on apoe−/−mice have revealed that TMAO reduces the expression of the Cyp7a1 and CYP27A1 enzymes in the traditional pathway of hepatic bile acid production, which is how it suppresses bile acid synthesis, thus accelerating aortic AS formation (Ding et al., 2018). Resveratrol inhibited trimethylamine production by remodeling gut microbiota, increased bile salt hydrolase activity, enhanced bile acid depolymerisation in mice, enhanced *de novo* synthesis of hepatic bile acids, and reduced TMAO-induced atherosclerosis (Chen et al., 2016).

#### Can TMAO be used as an independent predictor of stroke development and prognosis?

Studies have found that elevated plasma TMAO levels are associated with an increased risk of first stroke and are an important predictor of early neurological deterioration in patients with ischemic stroke (Rexidamu et al., 2019; Hou et al., 2020), directly affecting the area of cerebral infarction and adverse consequences after stroke (Zhu W. et al., 2021). In a case–control study conducted by Chen et al. (2022) and colleagues, large artery atherosclerotic (LAA) ischemic stroke group’s plasma TMAO levels were found to be considerably greater than those of the control group, after adjusting for traditional stroke risk factors. It shows that Plasma TMAO concentrations may be a potential biomarker for recurrent major vascular events, as the risk of recurrent major vascular events was increased by a factor of 2.128% in subjects with a LAA stroke and plasma TMAO levels above 126.83 pg/mL. Plasma TMAO levels must exceed 4.95 μmol/L to accurately predict moderate to severe stroke. However, other studies suggest that 6.6 μmol/L is the optimal concentration (Rexidamu et al., 2019).

A recent analysis of 256 patients with ischemic stroke revealed that increased plasma TMAO levels may be associated with post-stroke cognition (Zhu et al., 2020). In exploring the relationship between TMAO and neurological recovery after ischemic stroke, it was determined that TMAO promoted proliferation of ischemic stroke-responsive astrocytes and glial scar formation in middle cerebral artery occlusion/reperfusion rats, which worsened post-ischemic neurological damage (Su et al., 2021). To look into the relationship between TMAO levels and dysbiosis of the gut microbiota in patients with transient ischemic attack or large-artery atherosclerotic stroke, Yin et al. (2015) carried out case–control research in 2015. The findings demonstrated that rather than being greater than the asymptomatic group, patients with transient ischemic attack and stroke had considerably lower levels of TMAO than those with no symptoms. Similar findings were made by Skagen et al. (2016) in 2016: serum γBB and carnitine increased in patients with carotid atherosclerosis, but TMAO or TML did not increase. Therefore, it is debatable whether TMAO can be employed as a marker to determine the prognosis and severity of stroke as well as its function as a mediator in the pathogenesis of atherosclerosis. Future research may be necessary to confirm its role in detecting vascular disease.

### Fecal microbiota transplantation (FMT)

Fecal microbiota transplantation (FMT) is a method of directly modifying the intestinal microbiota of the recipient to normalize its composition and acquire therapeutic effects. Initially used to treat recurrent or refractory *Clostridium difficile* infection, it can now be used to treat not only gastrointestinal diseases but also extra-gastrointestinal conditions, such as cardiovascular disease (Wang et al., 2019). Currently, animal model research makes up most of the fecal microbiota transplantation in stroke. These studies imply a potential positive benefit of healthy donor FMT. The possible mechanisms are: (1) FMT produces a neuroprotective impact by lowering the production of pro-inflammatory bacteria and gut microbiota metabolites in the gut, as well as by attenuating inflammatory reactions and oxidative stress in the brain (Kang and Cai, 2017); and (2) improving stroke risk factors (e.g., hypertension, diabetes, and obesity) and decreasing stroke incidence.

FMT therapy significantly elevated levels of isobutyric acid, butyric acid and isovaleric acid. Our findings imply that neurological scores and infarct volume are inversely linked with acetate, valeric acid and especially with butyric acid. Therefore, modulating the gut microbiota to affect SCFA levels might be a potential treatment for ischemic stroke (Chen R. et al., 2019). Lee et al. (2020) used microbial sequencing and metabolomic analysis to show that fecal grafts of young mice contained higher levels of SCFAs and associated bacterial strains, *Bifidobacterium longum*, *Clostridium symbiosum*, *Prevotella faecalis*, and *Lactobacillus fermentum*. These relevant strains were implanted into aged post-stroke mice and reduced their neurological impairments and inflammation. Spychala et al. (2018) and colleagues transplanted the gut microbiota from young and old mice to each other. By fecal gavage, altering the microbiota of young mice to resemble that of old mice increased mortality and cytokine levels, whereas altering the microbiota of old people to resemble that of young people increased survival and recovery after MCAO. This suggests that fecal microbiota transplantation modifies the gut microbiota, reduces the inflammatory reaction, and provide neuroprotective.

FMT reduces the incidence of stroke by improving stroke risk factors. For instance, Sung et al. (2017) and Kim et al. (2018) administered fecal microbiota to two groups of obese donor mice via oral tube feeding: groups of normally supported donor mice and resveratrol supported donor mice. Glucose clearance was enhanced in mice that received fecal suspensions from resveratrol-fed donors compared to mice that received fecal suspensions from normal-fed donors. Vrieze and colleagues concluded that gut microbiota could be developed as a drug therapy to improve insulin sensitivity in people (Vrieze et al., 2012). These data lead us to conclude that FMT can be used to deliver beneficial substances.

The levels of SCFAs in plasma are affected by modified microbial mixes, which in turn impacts blood pressure homeostasis (Yang et al., 2020). Suez et al. (2018) compared the efficacy of autologous FMT and probiotic approaches to reconstitute mucosal microbial communities in mice and humans and discovered that autologous FMT helped to restore the human gut microbiota and host gut transcriptome rapidly and completely in comparison to probiotic supplementation FMT from HTN patients to a germ-free mouse model increased blood pressure and decreased the diversity and abundance of gut bacteria (Li J. et al., 2017). Interestingly, FMT from SHR to normal rats led to an increase in SBP in normal rats. In contrast, FMT from normal rats to SHR decreased the SBP of SHR (Adnan et al., 2017). Zhong et al. (2021) conducted a retrospective study comparing blood pressure before and after washed microbiota transplantation (WMT) in hypertensive patients treated with WMT. The result showed that WMT had a hypotensive effect on patients with hypertension, especially in patients treated with WMT through the lower gastrointestinal tract.

According to a clinical investigation with a 10-year follow-up, individuals with acute ischemic stroke who received intravenous thrombolysis on average lived roughly 1 year longer than those who did not receive the treatment (Muruet et al., 2018). Less than one-third of patients receive intravenous tissue plasminogen activator (TPA) treatment within the advised time period, despite the fact that the advantages of TPA for acute ischemic stroke are time-dependent (Saver and Levine, 2010). Therefore, it is essential to develop a safe and efficient treatment for stroke patients. Although current evidence suggests that FMT is a generally safe treatment with few side effects, the long-term outcomes of FMT have not been fully elucidated (Wang et al., 2019), and some comparative results have been observed, including a study in an animal model of stroke that demonstrated an increase in mortality after FMT (Vendrik et al., 2020). For stroke, only animal research and a small number of human studies have been completed or are being conducted, and substantial double-blind randomized controlled trials will be required to further clarify the role of FMT in stroke.

In addition to FMT, probiotics have been actively researched as a potential gut flora-related therapy to date. Probiotics are defined as “live microorganisms that, when given in sufficient quantities, are beneficial to the health of the host” (Hill et al., 2014). Lactobacillus, Bifidobacterium, Lactococcus, Streptococcus, and Enterococcus are the most widely used probiotics. The most researched probiotics out of these are strains of Bifidobacterium and Lactobacillus (Sarkar et al., 2016). Probiotic pretreatment was beneficial in reducing brain damage by 52% and was neuroprotective (Sun J. et al., 2016; Wanchao et al., 2018). It also considerably decreased the size of cerebral infarcts (Akhoundzadeh et al., 2018). Lowering cholesterol levels (Noda et al., 2010), reducing vascular endothelial dysfunction (Malik et al., 2018), controlling the expression of inflammatory factors (Primec et al., 2019), and influencing macrophage polarization (Mulhall et al., 2020) are mechanisms of possible direct processes (Toral et al., 2018). In addition, probiotics can also influence a number of factors associated with risk factors for stroke, including diabetes (Huda et al., 2021), obesity (Seganfredo et al., 2017), hypertension (Zhang et al., 2021), gut dysbiosis and pathogenic bacterial infections (Zhao et al., 2020; Tan et al., 2021). It is important to remember that not every probiotic used in clinical studies has had beneficial results. Probiotics have not been found to affect TMAO levels in peripheral blood as of yet, according to at least three research (Tripolt et al., 2015; Borges et al., 2019; Chen et al., 2021). Probiotics had no discernible impact on the plasma immune mediator concentrations, neutrophil and monocyte phagocytosis, or blood culture response to immune stimulation in a randomized controlled trial of LGG + BB-12 (1.3–1.6109 CFU per day) treatment in elderly people living in care homes (Castro-Herrera et al., 2021). Probiotics did not enhance the intestinal flora’s dysbiosis in Si Chen’s study (Chen et al., 2021). They also did not increase the species diversity of the intestinal flora. It has also been demonstrated that some probiotic strains can worsen the inflammatory response by inducing M1 macrophage polarization (Guha et al., 2019) and increasing the production of pro-inflammatory cytokines.

The reasons for the differences in probiotic treatment may be that (1) the type of probiotic strain, the dose, and the length of the treatment all have a significant impact on the treatment’s efficacy. The majority of current clinical probiotic trials combine different probiotic strains, however the effects of microorganisms are incredibly varied and frequently strain-specific. The ideal strain combinations and time of treatment cannot be determined directly due to a dearth of research, which merits further thorough investigation. (2) Inconsistency and ambiguity within the research population. We think that variable outcomes with the same strain and dose of probiotic treatment may be caused by the diversity in gut flora composition across persons as well as the notable differences between rodent and human microbial communities. Although probiotics’ positive effects have shown some promise in research involving animals like mice and piglets, larger randomized double-blind human trials are still required, much like FMT. (3) The absence of more thorough tests. The reported randomized controlled trials have demonstrated methodological variability and relatively small sample numbers. Numerous studies have also neglected to properly analyze their patients, using solely self-reported symptomatological data.

### Challenges in the clinical application of gut microbiota in stroke

The clinical use of gut microbiota in stroke shows enormous potential, but there are still many obstacles to overcome before it can be effectively used in the clinic. Firstly, it’s unclear exactly how the mechanics work. The majority of the present research on gut microbiota and stroke is conducted on animals, and clinical trials are considerable scarcity. It has been established that the conclusions reached in animal investigations do not always translate into human therapeutic trials (Roberts et al., 2002). Therefore, it is unknown whether the limited mechanistic insights that have so far been made can be applied to humans. Secondly, there is the general lack of security. Defining a healthy microbiota is extremely difficult due to significant individual differences in the gut microbiota, which are influenced by a confounding combination of genetics, environment, chronic stress, antibiotic use, behavior, diet, and age. Careful testing and donor screening should be considered to minimize safety concerns with FMT, especially for multi-drug resistance (Ng et al., 2020). To improve the clinical efficacy of FMT, more extensive studies are required to identify single strains or microbial agents with specific benefits for stroke (Frye et al., 2015), so as to identify target populations for microbial therapeutic interventions, such as the optimal disease stage and age of the patient, and to evaluate the ability of each strain to remain viable and effective at a given target (Belizário and Napolitano, 2015). The final challenge is the lack of specificity. One potential factor in the failure of antibiotic therapy is bacterial resistance (Yan et al., 2015). In addition, a lack of specificity in antibiotics is the root cause of antibiotic resistance to post-stroke infections. After a stroke, a number of biological processes in the body are disturbed, including bacterial translocation, disruption to the intestinal barrier, and changes to the gut microbiota. Antibiotics can therefore be used in conjunction and focus on the particular causes of infection susceptibility after stroke as a “symptomatic but not curative” treatment. In the future, we anticipate customizing FMT for various individuals and under different circumstances based on the host and disease.

## Conclusion

This study summarizes and predicts current research hotspots and future directions with the aid of scientometrics and visual analysis to show the current status and trends of research on the relationship between gut microbiota and stroke. It also gives researchers in related fields a better understanding of the association between gut microbiota and stroke through analysis of the literature. Summarized as follows:

(1) A bibliometric approach was used to describe the distribution of national institutional and disciplinary journals and authors in the study of stroke and gut microbiota correlations.
(2) The co-occurrence analysis of keywords and references revealed that inflammation and immunity, bacterial metabolites, and fecal microbiota transplantation are the hotspots of study in this field.
(3) The dynamic evolution of the mechanisms in this emerging field from 2002 to 2021 is summarized.
(4) The current challenges and corresponding solutions for the study of stroke and gut microbiota correlations.

In conclusion, research on gut microbiota and how it affects health and illness is a relatively new and quickly developing area of biology and medicine. Diseases that provide a risk for stroke and post-ischemic consequences may have gut microbiota as a possible treatment target. In order to improve the clinical application of gut microbiota in stroke disease, we must get through the obstacles we are now facing at this stage.

## Author contributions

SH: conceptualization, methodology, writing—original draft preparation, manuscript, and figure preparation. LC: conceptualization, methodology, writing—original draft preparation, manuscript, and software. PC: writing—review and editing. WK: supervision. All authors have read and agreed to the published version of the manuscript.

## Conflict of interest

The authors declare that the research was conducted in the absence of any commercial or financial relationships that could be construed as a potential conflict of interest.

## Publisher’s note

All claims expressed in this article are solely those of the authors and do not necessarily represent those of their affiliated organizations, or those of the publisher, the editors and the reviewers. Any product that may be evaluated in this article, or claim that may be made by its manufacturer, is not guaranteed or endorsed by the publisher.
